# Pensions Problems

**Published:** 1919-08-02

**Authors:** 


### PENSIONS PROBLEMS.

With every desire to see legitimate grievances redressed and every sympathy with those who seek to jog Government Departments out of the comfortable lethargy which seems to characterise the servants of the State in any "nationalised" industry, it is yet impossible to support the philippics which the Medical Correspondent of the *Times* has recently directed against the medical branch of the Pensions Ministry. His criticisms are, many of them, patently unfair; and his constructive suggestions as to what ought to be, or ought to have been done, betray either bias or profound ignorance. The result of ill-judged attacks, such as those mentioned and that of Marshal Sir D. Haig, which we commented upon not long ago (The Hospital, July 5, 1919, p. 339) is that the Ministry refutes them easily and is encouraged to regard itself as immaculate.

Let us take one of the subjects for which the Pensions administration has been criticised, what may be called the demobilisation assessment. Many of our readers are probably aware that on demobilisation from war service the soldier has to fill up before leaving his unit a form known as Z 22. This form places two alternative courses before the soldier: he can either sign a statement that he is (at the moment) suffering no disability for which he wishes to claim a pension, or he can sign a statement that he is so suffering, in which case he is invited to furnish details of his disabilities and to be examined by a medical officer of the R.A.M.C. before being demobilised. It is to be noted that to sign the form Z 22 as having no disability and making no claim does not estop a soldier from putting in a claim subsequently if he finds that some disability does later on arise (due to war service), although he was not cognisant of it on demobilisation. An old healed wound may break out again, an aneurysm may develop, a relapse of an apparently cured infective illness may take place; all these or similar troubles entitle a man to reopen the matter of his claim to a pension, in spite of his having signed a statement of "no disability" on demobilisation.

The contention of the *Times* is that this system is vicious. It is contended that the soldier should not be asked whether or not he claims a pension, but should be submitted to medical examination as a routine before demobilisation, so that an expert opinion may be available on the point. And it is further sought to buttress this proposal by the argument that the regimental medical officer, to whom the duty of making these examinations would fall, has the great advantage of knowing all about the men's previous illnesses and medical history, and is thus the most competent to pass judgment on their (pensionable) condition. The answer to this argument is simple and crushing. In the first place, such a routine examination was impossible, without so delaying the demobilisation as to have aroused bad feeling and recrimination, of which there was already more than enough in evidence—and not altogether unjustly. It would have seriously retarded the demobilisation of the R.A.M.C. officers, who were already seriously in arrears of demobilisation compared with the rest of the Army, and were also urgently needed at home to cope with the influenza epidemic. Nor would it have been likely to disclose any cases of disability other than those which were in fact detected by the Z 22 scheme.

The regimental medical officer knows and can know practically nothing of the past medical history of his men. His battalion, during fighting such as that which characterised the last few months of the war, is renewed, practically from colonel to drummer boy, about once every two or three months. His sick and wounded are evacuated from under his care—all he can do for them is first-aid in most cases—within a few hours of their being unfit for fighting; and he never sees them again in nineteen cases out of twenty. The nature of his work fits him far less than that of base hospitals or C.C.S. for estimating the severity of injuries and diseases, or their probable after-effects. Finally, by the fourth year of the war it was widely, though not universally, recognised and acted on in the British Army that the proper place for the best educated and most experienced doctors was *not* the firing-line but a medical unit further back. It followed that the average regimental medical officer tended to be selected from amongst the youngest, or least experienced, or least highly educated of the doctors. All these considerations would occur at once to those with active service experience, but they do not seem to have struck the *Times* correspondent.

